# Directly Printed Embedded Metal Mesh for Flexible Transparent Electrode via Liquid Substrate Electric‐Field‐Driven Jet

**DOI:** 10.1002/advs.202105331

**Published:** 2022-03-01

**Authors:** Zhenghao Li, Hongke Li, Xiaoyang Zhu, Zilong Peng, Guangming Zhang, Jianjun Yang, Fei Wang, Yuan‐Fang Zhang, Luanfa Sun, Rui Wang, Jinbao Zhang, Zhongming Yang, Hao Yi, Hongbo Lan

**Affiliations:** ^1^ Shandong Engineering Research Center for Additive Manufacturing Qingdao University of Technology Qingdao 266520 China; ^2^ Key Lab of Industrial Fluid Energy Conservation and Pollution Control, Ministry of Education Qingdao University of Technology Qingdao 266520 China; ^3^ Shien‐Ming Wu School of Intelligent Engineering South China University of Technology Guangzhou 511442 China; ^4^ School of Information Science and Engineering and Shandong Provincial Key Laboratory of Laser Technology and Application Shandong University Qingdao 266327 China; ^5^ State Key Laboratory of Mechanical Transmission Chongqing University Chongqing 400044 China

**Keywords:** electric field driven jet, flexible transparent electrodes, liquid substrate, metal mesh, microscale 3D printing

## Abstract

Flexible transparent electrodes (FTEs) with embedded metal meshes play an indispensable role in many optoelectronic devices due to their excellent mechanical stability and environmental adaptability. However, low‐cost, simple, efficient, and environmental friendly integrated manufacturing of high‐performance embedded metal meshes remains a huge challenge. Here, a facile and novel fabrication method is proposed for FTEs with an embedded metal mesh via liquid substrateelectric‐field‐driven microscale 3D printing process. This direct printing strategy avoids tedious processes and offers low‐cost and high‐volume production, enabling the fabrication of high‐resolution, high‐aspect ratio embedded metal meshes without sacrificing transparency. The final manufactured FTEs with 80 mm × 80 mm embedded metal mesh offers excellent optoelectronic performance with a sheet resistance (*R*_s_) of 6 Ω sq^−1^ and a transmittance (*T*) of 85.79%. The embedded metal structure still has excellent mechanical stability and good environmental suitability under different harsh working conditions. The practical feasibility of the FTEs is successfully demonstrated with a thermally driven 4D printing structure and a resistive transparent strain sensor. This method can be used to manufacture large areas with facile, high‐efficiency, low‐cost, and high‐performance FTEs.

## Graphical abstract

A facile fabrication method for flexible transaprent electrodes (FTEs) with embedded metal mesh is proposed by using liquid substrate electric‐field‐driven (LS‐EFD) microscale 3D printing process. The fabricated FTEs exhibit excellent photoelectric properties, remarkable mechanical stability and environmental adaptability. The practical feasibility of the FTEs is successfully demonstrated with a thermal‐driven 4D printing structure and a resistive transparent strain sensor.

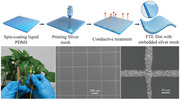

## 1 Introduction

Flexible transparent electrodes (FTEs) with excellent photoelectric properties, good stability, and high flexibility are critical and indispensable components for next‐generation flexible optoelectronic devices.^[^ ^1^, ^2^, ^3^, ^4^, ^5^, ^6^ ^]^ Indium tin oxide (ITO) is widely used as a conductive transparent material, but it is difficult to be used in FTEs due to its intrinsic brittleness, scarcity, and high‐temperature magnetron sputtering process.^[^ ^7^, ^8^ ^]^ Researchers have recently developed a variety of alternative conductive materials to replace ITO, including carbon‐based materials,^[^ ^9^, ^10^, ^11^, ^12^, ^13^, ^14^ ^]^ conductive polymers,^[^ ^15^, ^16^ ^]^ metal‐based materials,^[^ ^17^, ^18^, ^19^, ^20^, ^21^, ^22^, ^23^, ^24^ ^]^ and hybrid materials.^[^ ^25^, ^26^, ^27^ ^]^ Of these, common issues in FTEs using carbon‐based materials and conductive polymers include poor electrical conductivity and poor environmental stability, which limit their applications in flexible optoelectronics.^[^ ^28^ ^]^ Metals are the most promising candidate material for FTEs. They offer high conductivity as well as high yet tunable mechanical flexibility. However, each material also has unique advantages and critical challenges.^[^ ^29^, ^30^, ^31^ ^]^ For example, Schubert et al.^[^ ^32^ ^]^ fabricated an electrode structure with an *R*_s_ of 19 Ω sq^−1^ and *T* of 83% at 580 nm wavelength by thermally evaporating an ultrathin metal film that is superior to 32 Ω sq^−1^ of ITO film in this spectral range. Although the ultrathin metal film has excellent electrical conductivity and good optical transmittance, it requires complicated processing such as thermal evaporation and sputtering deposition during the manufacturing process. It is difficult to solve the contradiction of simultaneous increases in transmittance and conductivity. Metal nanowire networks offer facile and full‐solution processing as well as excellent ductility. They also have poor chemical stability, poor adhesion to the substrate, and high contact resistance that hinders extensive industrial applications. The electrical and optical properties of FTEs based on metal meshes can be optimized and regulated by simply changing the line width, pitch, aspect ratio (AR), shape, and mesh type in metal meshes. Thus, FTEs based on metal meshes have been considered promising alternatives to ITO and have shown broad application prospects in many applications.^[^ ^33^, ^34^, ^35^, ^36^ ^]^

In general, a metal mesh embedded in a flexible transparent substrate is far better than an embossed metal mesh in terms of surface roughness, chemical stability, environmental stability, and mechanical fatigue strength.^[^ ^37^ ^]^ Therefore, the manufacturing of embedded metal meshes has attracted extensive attention. Nevertheless, the manufacturing of high‐performance FTEs with embedded metal meshes remains a major challenge. Most methods utilize hybrid manufacturing. For example, Li et al.^[^ ^38^ ^]^ fabricated FTEs with an embedded metal mesh with a value of *T* over 90% and an *R*_s_ less than 1 Ω sq^−1^ by combining lithography, electroplating, and imprint transfer. This material was successfully applied to a transparent and flexible thin‐film heater. Chen et al.^[^ ^39^ ^]^ adopted ultraviolet nanoimprint lithography technology to fabricate flexible metal mesh electrodes without contact junctions. This material has better flexibility than a commercial polyethylene terephthalate (PET)/ITO. The fabricated PET/polydopamine (PDA)@Ag grid‐based polymer solar cells yield a power conversion efficiency of 11.6%. Cui et al.^[^ ^40^ ^]^ produced an embedded copper mesh FTEs with an *R*_s_ of 0.03 Ω sq^−1^ and *T* of 86% via nanoimprint, scraping, and electroplating. The application was automotive windshield deicing. Although the current hybrid manufacturing process can manufacture high‐performance embedded metal mesh FTEs, it is difficult to remove photolithography, electroplating, laser direct writing, crack template, vacuum evaporation, nanoimprinting, and other processes. This makes the entire process more complex with high production costs and environmentally toxicity.^[^ ^41^, ^42^, ^43^, ^44^, ^45^, ^46^, ^47^ ^]^ Our previous work^[^ ^48^ ^]^ demonstrated a template‐free and plating‐free fabrication method of embedded metal mesh FTEs with a *T* of 85.79% and *R*_s_ of 0.75 Ω sq^−1^. However, it still needs to be combined with microscale 3D printing and hot embossing technology—this makes the process even more complicated. Printing technologies offer direct rapid prototyping, which is often considered a green manufacturing process. Lewis and Wood^[^ ^49^ ^]^ proposed an embedded 3D printing method that directly extrudes conductive ink into a liquid rubber substrate through a printing nozzle. This produced good application effects in tensile sensors. However, it is difficult to produce high‐resolution wires via direct extrusion. Song et al.^[^ ^50^ ^]^ fabricated an embedded conductive circuit with a resistance of 360 Ω by inkjet printing AgNP ink into viscous liquid polydimethylsiloxane (PDMS). However, inkjet printing technology is only applicable to conductive ink with viscosity values below 30 cp; it cannot achieve printing of conductive pastes with high solid contents and high viscosity, thus making the conductivity of printed lines poor. Although several cost‐effective manufacturing methods for embedded metal meshes have been developed based on photolithographic plating, nanoimprinting, inkjet printing, electrohydrodynamic jet printing, and hybrid manufacturing processes, the performance of such manufactured and embedded metal meshes remains lower than conventional manufacturing processes. Thus, low‐cost, simple, and efficient manufacturing of high‐performance embedded metal mesh FTEs is still an important challenge.

We have demonstrated the application capability of electric‐field‐driven microscale 3D printing technology in our previous work.^[^ ^51^, ^52^ ^]^ In this work, we propose a new method for low‐cost and simple fabrication of embedded metal mesh FTEs via a newly developed liquid substrate electric‐field‐driven (LS‐EFD) microscale 3D printing. First, a layer of liquid material was spin coated on the surface of the flexible substrate, and then an ultrathin metal mesh with a large AR was printed directly on the liquid film substrate using LS‐EFD microscale 3D printing technology. Finally, the embedded metal mesh FTEs with excellent photoelectric performance was obtained through low temperature sintering. Due to the inhibition of the liquid film substrate, this method can directly encapsulate the high resolution and high AR metal mesh in a flexible substrate, which requires no additional packaging processes. The presence of the liquid film substrate makes it possible to achieve stable jet printing by applying a low voltage. To best our knowledge, there is no research has been conducted to demonstrate the phenomenon and results of electrohydrodynamic jet printing on liquid substrates. In this paper, we have developed an innovative approach to the direct encapsulation and fabrication of flexible transparent conductive films with embedded metal meshes using liquid PDMS as the print substrate, which brings a broad research field for electrohydrodynamic jet technology. The embedded metal structure not only improves the FTEs's chemical stability in harsh environments but also improves the mechanical stability under high bending stress. This direct printing strategy avoids tedious processes and offers low‐cost and high‐volume production, enabling the fabrication of high‐resolution, high‐AR embedded metal meshes without sacrificing transparency. The fabricated FTEs exhibits superior optical and electrical properties versus previous fabrication methods.

## 2 Results and Discussion

**Figure 1:**
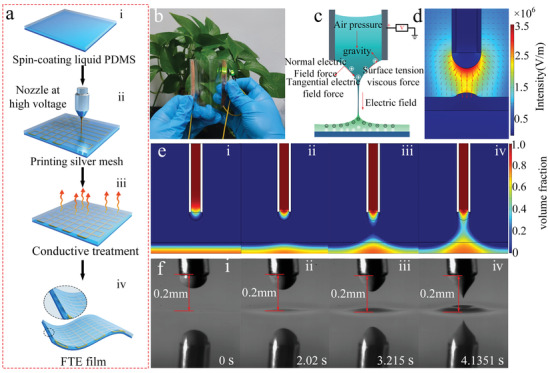
a) Schematic diagram of manufacturing principle of embedded metal mesh FTE. b) Macroscopic image of the of FTE with embedded metal mesh. c,d) The schematic and numerical simulation of LS‐EFD microscale 3D printing. e) Numerical simulation diagram of Taylor cone of nozzle and needlepoint structure of liquid substrate formation process. f) Taylor cone of nozzle and needlepoint structure formation process of liquid PDMS substrate captured by a high‐speed camera.

PDMS was selected as the material of liquid film substrate in this work due to its good stretchability, strong chemical stability, and high optical transmittance. The manufacturing process of the FTEs with an embedded metal mesh is shown in **Figure** **1**a and includes three main steps: i) spin coating a layer of liquid PDMS on the PET substrate; ii) metal mesh printing using the LS‐EFD microscale 3D printing process (Movie S1, Supporting Information); and iii) conductive treatment of the embedded metal mesh. The final fabricated FTEs with an embedded metal mesh is shown in Figure 1b. It has an area of 80 mm × 80 mm and a pitch of 1 mm. Notably, the FTEs with an embedded metal mesh not only has good flexibility but also has excellent optical transmittance. The LS‐EFD microscale 3D printing technology is a novel printing method (see Figure 1c). During the printing process, the printed materials are pushed toward the tip of the nozzle to form a meniscus under the combined action of air pressure and gravity. When the printing nozzle is close to the liquid PDMS, the polarization phenomenon will occur under the action of the external electric field, which is reflected by an overall negative polarization charge on the upper surface of the PDMS and an overall positive polarization charge on the lower surface. The printed material will form a very fine jet deposited on the printed substrate when the electric field force breaks through the surface tension of the printed material (see more details in Figure S1, Supporting Information). The electric field intensity between nozzle and PDMS liquid film substrate is enhanced due to the generation of polarized charge. This in turn leads to formation of a liquid needlepoint structure on the surface of PDMS liquid film substrate. To demonstrate the electrostatic induction between the printing nozzle and the liquid needlepoint structure, a numerical model of the electric field intensity distribution in a 2D space was performed via COMSOL multiphysics simulation software (Figure 1d). The geometric model and meshing results are shown in Figure S2 in the Supporting Information. The numerical simulation results show that the electric field lines emanating from the tip of the nozzle are more concentrated on the surface of the liquid needlepoint structure, thus greatly enhancing the electric field strength between them. This improves the electric field's ability to confine the print jet, thus stabilizing the printing. To verify the correctness of the printing principle model, a complete cone jet injection process was simulated via the multiphysics simulation software COMSOL, as shown in Figure 1e,i–iv. The volume fraction of 1 in the red area represents the printed material, and the volume fraction of 0 in the blue area represents air. Figure 1e,i,iv shows the initial injection state and the final injection state, respectively. The formation process of liquid needlepoint structure and the process of printing material gradually elongates to produce a jet injection (Movie S2, Supporting Information). The numerical simulation results show that the formation of the liquid needlepoint substrate not only effectively shortens the printing height but also enhances the constraint ability of the electric field force on the cone jet. In order to show the process of jet printing more intuitively and clearly, the liquid needlepoint formation process was captured by a high‐speed camera as shown in Figure 1f,i–iv (Movie S3, Supporting Information). The results are consistent with the numerical simulation results and further verify the accuracy and feasibility of the printing principle.

PDMS is a carrier of the metal mesh and its thickness was a primary consideration. PDMS films of different thicknesses were first obtained by controlling the speed and time of the spin coater (Figure S3, Supporting Information). Experimental studies found that the required drive voltage decreased with increasing PDMS thickness when different thicknesses of PDMS films were used as a printing substrate (Figure S4, Supporting Information). This is because the thicker the PDMS, the less resistance the internal material creates to fluid movement on the upper surface, resulting in greater fluidity. Under the same electric field action, there is enough liquid PDMS to supplement the formation of the liquid needlepoint structure, which is more likely to produce a larger liquid needlepoint structure (Figure S5, Supporting Information). The existence of the liquid needlepoint structure further shortens the printing height, increases the restraint effect of the electric field on the Taylor cone jet, and improves the stability of printing. In addition, the encapsulation of the printed conductive paste by the liquid film limits its further spreading, improving the resolution of the printed silver lines while enabling a one‐step embedding of the silver lines. The microstructure of the silver wire is shown in Figure S6 in the Supporting Information. The reduction of driving voltage also represents the choice of smaller models of power supply in actual production, which has a significant cost advantage in large‐scale production. The degree of curing of the PDMS determines the successful manufacture of fully embedded metal meshes and semi‐embedded metal meshes. Thus, PDMS films with different curing degrees were obtained by adjusting the precuring temperature and time before printing the metal mesh. Figures S7 and S8 in the Supporting Information are the minimum driving voltage and needlepoint of the liquid substrate corresponding to PDMS at different curing degrees, respectively. The results show that the minimum driving voltage is further increased with increasing degrees of PDMS curing. A higher PDMS curing degree makes it more difficult for the needlepoint of the liquid substrate to form, which in turn leads to a weaker electric field focusing effect and a larger driving voltage. The surface energy and rheological properties of PDMS also are changed due to different degrees of crosslinking curing when PDMS is heated. This affects the printing results for the silver wire as shown in Figure S9 in the Supporting Information. The printing process of PDMS in different states was captured by a high‐speed camera as shown in Figure S10 in the Supporting Information. It is worth noting that liquid PDMS substrates can be used not only as part of a metal mesh carrier substrate for the fabrication of FTEs but also as a sacrificial material for the stable printing of high resolution metal meshes on other rigid substrates. Considering the factors such as print speed and the coating of liquid substrates, it is possible to develop a parallel printing method with multiple printheads to improve printing efficiency, which is our future work. Furthermore, during the microscale 3D printing process of metal meshes, the driving voltage, printing speed, and air pressure value of the printing system determine the quality of the formed dimensional shape of the metal mesh. The optimal process parameters were determined through a series of experiments, and the results show that the silver lines have the best microstructure and resolution when the thickness of the liquid PDMS is 10–60 µm, the driving voltage is 300–380 V (see Figure S11, Supporting Information), the printing speed is 20–80 mm s^−1^ (see Figures S12 and S13, Supporting Information), and the air pressure value is 180–240 kPa (see Figures S14 and S15, Supporting Information).

**Figure 2:**
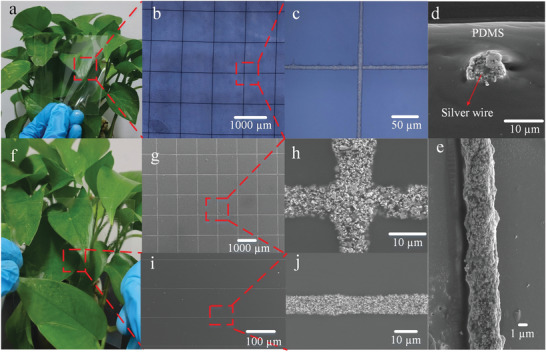
Macroscopic image of the FTE with fully embedded metal mesh. b,c) Micro image of the FTE with fully embedded metal mesh. d) SEM diagram of fully embedded silver wire. e) SEM enlargement of single silver wire. f) Macroscopic image of the FTE with semi‐embedded metal mesh. g,h) SEM images of the semi‐embedded metal meshes and a partially enlarged SEM image. i,j) SEM images of the semi‐embedded silver stripe and a partially enlarged SEM image.

**Figure** **2** shows representative optical images of fully embedded (Figure 2a–e) and semi‐embedded metal meshes (Figure 2f–j). Figure 2a is a macroimage of fully embedded metal mesh FTEs with an area of 80 mm × 80 mm, pitch of 1000 µm, and a line width of 6.7 µm. The green vegetation can be clearly observed at the normal distance of human eyes, thus indicating that the fabricated embedded metal mesh has good optical transmittance. Figure 2b,c shows the microstructure and partially enlarged details of the metal mesh, respectively. Figure 2d is the scanning electron microscopy (SEM) of silver wire cross‐section after sintering at 130 °C for 1 h. The silver wire is completely embedded in the PDMS film, and the AR of the silver wire can reach 0.77. To observe the microscopic morphology of the metal mesh more clearly, the fabricated embedded metal meshes were also immersed in a solution of propylene glycol monomethyl ether acetate containing 1% tetrabutyl ammonium fluoride to dissolve the cured PDMS. Figure 2e shows the SEM image structure of the silver line after PDMS was dissolved. It can be seen that when the printed silver line was completely embedded in the PDMS, it can still maintain a complete line structure and have a good morphology. A semi‐embedded metal mesh FTEs was fabricated on liquid PDMS cured at 90 °C for 6 min as shown in Figure 2f. Here, Figure 2I,j is SEM images and partially enlarged details of the semi‐embedded silver stripe, respectively. Figure 2g,h shows the SEM image and partially enlarged details of semi‐embedded metal mesh, respectively. The results show that both full embedded and semi‐embedded metal mesh FTEs have good microstructure and morphology characteristics.

**Figure 3:**
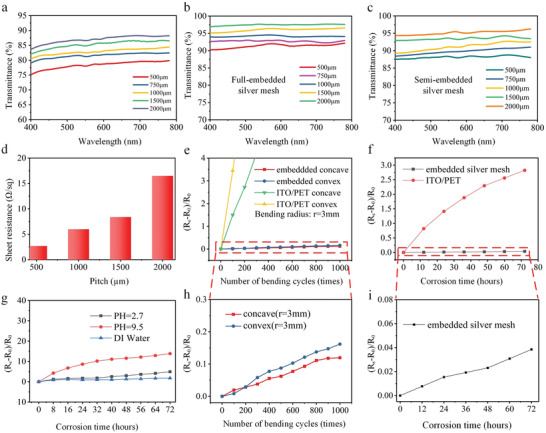
Optical performance, mechanical stability, and environmental stability of FTE: a) Optical transmittance of fully embedded metal mesh (width: 10 µm) with PDMS/PET substrate with different pitch sizes in the visible range. b) Optical transmittance of fully embedded metal mesh (width: 10 µm) without PDMS/PET substrate with different pitch sizes in the visible range. c) Optical transmittance of semi‐embedded metal mesh (width: 13 µm) without PDMS/PET substrate with different pitch sizes in the visible range. d) *R*_s_ of fully embedded metal mesh at different pitches. e,h) Comparison of bending stability between ITO/PET and embedded metal mesh with bending radius of 3 mm. f,i) Damp heat test comparison between embedded metal mesh and ITO/PET film. g) Chemical stability test of embedded metal mesh.

The photoelectric properties are important indicators for FTEs. **Figure** **3**a–c compares the optical transmittance of a fully embedded metal mesh with a PDMS/PET substrate (Figure 3a), a fully embedded metal mesh without PDMS/PET substrate (Figure 3b), and a semi‐embedded metal mesh without PDMS/PET substrate (Figure 3c). The silver wire widths of fully embedded and semi‐embedded are 10 and 13 µm, respectively, and the pitch ranged from 500 to 2000 µm. The optical transmittance (at *λ* = 550 nm, pitch = 1000 µm) of fully embedded and semi‐embedded metal mesh without PDMS/PET substrate is 92.85% and 91.09%, respectively. Compared with those with PDMS/PET substrate, the optical transmittance of fully embedded and semi‐embedded metal meshes increased by 9.91% and 9.19%, respectively. Figure 3d shows the variations in *R*_s_ with the pitch of the embedded silver mesh. The results show that the *R*_s_ of FTEs increases with the increase of pitch, while the optical transmittance increases gradually, indicating that the manufactured FTEs has a good trade‐off between optical transmittance and *R*_s_. A metal mesh (area: 80 mm × 80 mm, line width: 10 µm, pitch: 1000 µm) achieved *R*_s_ of 6 Ω sq^−1^ and *T* of 92.85%, indicating that the fabricated flexible FTEs has excellent photoelectric properties. Mechanical stability and environmental adaptability are other important metrics for evaluation. Figure 3e,h shows the comparison bending test results of the embedded metal mesh and the commercial FTEs with ITO/PET prepared in this work. After a bending test with a bending radius of 3 mm and bending cycle of 1000 times, the *R*_s_ change rates of convex bending and concave bending are 0.16 and 0.12, respectively, showing excellent bending resistance compared with ITO/PET FTE. In addition, a 72 h damp heat test (temperature: 85 °C and humidity: 85%) was performed to determine whether the manufactured FTEs was still serviceable after being subjected to high temperature and humidity; the test results are shown in Figure 3f,i. After the damp heat test, the *R*_s_ changes the rate of FTEs manufacturing and is only 3.85%. Here, the *R*_s_ change rate of the ITO/PET film reaches 283%, thus indicating that it can be used in harsh environments. To further explore the environmental adaptability of embedded metal mesh FTEs, the FTEs was also soaked in solutions at pH 2.7, 9.5, and 7.0 to simulate different chemical environmental conditions. The variation of *R*_s_ was measured every 8 h. The results show the *R*_s_ change rates of the FTEs in acidic solution, alkaline solution, and neutral solution are 5%, 13.8%, and 2%, respectively, after soaking for 72 h. This further confirms that our FTEs has excellent chemical stability. Thus, these results indirectly prove that the FTEs fabricated in this work have excellent comprehensive performance.

**Figure 4:**
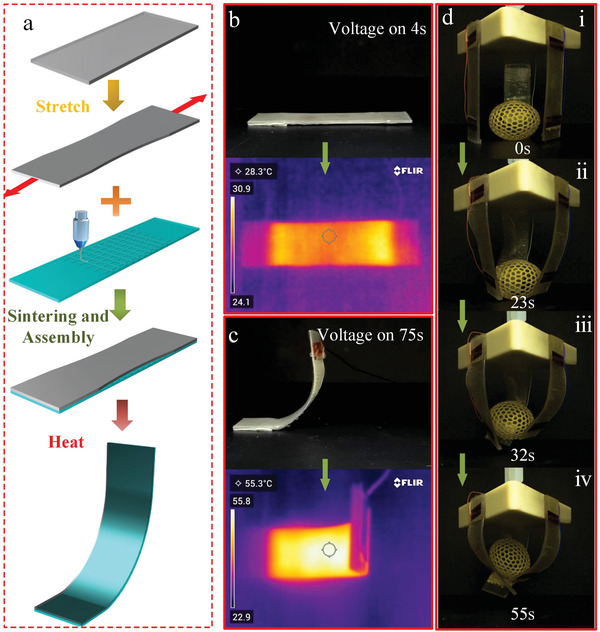
a) Schematic diagram of the 4D printing process. b,c) 4D printed sample deformation process and corresponding thermal imaging. d–g) A grasping device based on 4D printing.

4D printing technology has become a current and popular topic. It adds the 4D “time” to the 3D printing structure to achieve the shape deformation of the 3D structure by changing the external environment such as thermal, electric, optical, and magnetic conditions.^[^ ^53^, ^54^, ^55^ ^]^ Thermally responsive shape‐memory polymers (SMPs) have become one of the most exciting materials due to their fast response time, large deformability, and high compatibility with multimaterial 3D printing. Currently, the most commonly used heating method for SMPs‐based 4D printing is external heating, however, this heating method cannot accurately control the heating temperature of 4D printing structure, which hinders the further application of 4D printing. Indeed, the effective heating method is key to controlling the “time” dimension.^[^ ^56^, ^57^, ^58^ ^]^ Here, we successfully applied a fabricated FTEs to a thermally driven 4D printed structure, this approach achieved a thermal drive capabilities for 4D printing. The transparent electrode structure had no negative visual impact from the 4D printed structure. The schematic diagram of the basic realization process is shown in **Figure** **4**a. The thermally responsive SMPs was first heated and stretched, and then the FTEs was attached to one side of the SMPs through a very high bond (VHB) tape. A voltage of 5 V is connected to the FTEs lead, and infrared thermal image of the initial state is shown in Figure 4b. When the heating temperature is higher than the glass transition temperature of SMPs, the heated region of the SMPs sample will produce shrinkage deformation, and the conglutinated FTEs could not be compressed—this leads to a deformation including a bent shape following strain mismatch in the heated area of SMPs sample (Figure 4c and Movie S4, Supporting Information). The heating schematic diagram of the embedded metal mesh FTEs is shown in Figure S16a in the Supporting Information and the thermal response diagram is shown in Figure S16b in the Supporting Information. Figure S17 in the Supporting Information shows thermal distribution images at different time periods under a 2 V DC voltage, which demonstrates good and stable heating performance. Furthermore, a grasping device based on 4D printing was composed of three graspers (SMPs samples) and successfully grasped an elliptical sphere with a hollow structure as shown in Figure 4d,i–iv (Movie S5, Supporting Information). The successful application of FTEs based on embedded metal mesh in 4D printing proves that our embedded metal mesh has good application potential and feasibility.

**Figure 5:**
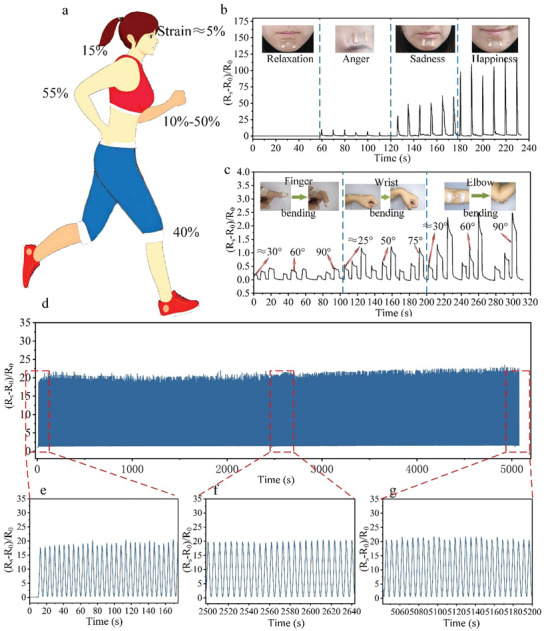
a) Stress variables in different positions of the body. b) The strain sensors corresponding to different facial expressions responded in real time. c) The corresponding strain sensor of human joints responds in real time under different bending angles. d) Fatigue test of the strain sensors.

Traditional strain sensors are generally made of metal or semiconductors and are usually used to detect strain deformation of rigid structures less than 5% such as bridge detection, track vibration deformation, and other fields.^[^ ^59^, ^60^, ^61^ ^]^ Wearable strain sensors offer convenience, good skin adhesion, and high sensitivity. They exhibit tremendous applications including heart rate monitoring, blood pressure and pulse, and human motion signal monitoring.^[^ ^62^, ^63^, ^64^ ^]^ Conventional fabrication methods are mostly used to fabricate strain sensors with rigid substrates and there are still huge challenges in the fabrication of flexible and transparent strain sensors including low efficiency and high cost. These cannot achieve flexible and transparent functions. Flexible and transparent strain sensors have unique advantages in terms of good visibility and ease of observation. These can help better observe detected areas when performing signal data acquisition. There are many manufacturing methods for flexible strain sensors including chemical vapor deposition, magnetron sputtering, vacuum evaporation coating, solution‐process, and self‐assembly process.^[^ ^65^, ^66^, ^67^, ^68^ ^]^ However, in addition to the self‐assembly process and solution‐process, the above manufacturing technologies or hybrid manufacturing technologies are complicated, costly and environmentally unfriendly. This limits further development of high‐performance flexible transparent strain sensors. To further prove the application ability of the FTEs manufactured, we designed a resistive flexible transparent strain sensor with Ecoflex/PDMS composite structure with a large range of withstand strains through LS‐EFD microscale 3D printing technology. Furthermore, to verify the feasibility of the scheme, the fabricated FTEs with Ecoflex and PDMS composite structure was fitted on the wrist, as shown in Figure S18 in the Supporting Information. The corresponding thermal image of the wrist at different bending angles shows excellent heating performance. **Figure** **5**a shows the strain variables at different positions in the human body. A flexible transparent strain sensor was manufactured using a low‐temperature sintered conductive silver paste printed with ultrafine embedded metal mesh for monitoring of human movement. The resistance strain sensor has a maximum gauge factor (GF) of more than 5000 and could monitor small deformation movements in the strain range of up to 5% and can respond quickly. The resistance strain sensor was attached to the human face (chin and forehead), and changes in different emotions could be monitored by changes in the resistance value, e.g., relaxation, anger, sadness, and happiness (Figure 5b). The results showed that no signal was generated when the human body was in a quiet and relaxed state; conversely, the resistance change rate was the largest when the human body was in a happy state. To monitor the large bending deformation of human joints, a strain sensor made of stretchable silver paste was used to achieve a GF value of 33.33 when the strain value was above 60%, as shown in Figure 5c. The results showed that the resistance change rate was different at different bending angles: The resistance change rate was the largest when the bending angle reached 90°. To verify the fatigue characteristics and durability of the fabricated flexible transparent strain sensor, we next tested the resistance change of the strain sensor at a stretch rate of 60% and a cycle of 1000 times. Figure 5d shows that the strain sensor still has good stability after 1000 tensile cycles. This demonstrated its good potential for practical application. The strain sensor offered good flexibility and optical transparency without affecting the optical transmittance. It can nicely adhere to the human skin and achieve arbitrary stretching, twisting, and bending functions, thus enabling accurate measurements. The application of this technology further verifies the feasibility and universality of our proposed process method as well as the good application potential.

## 3 Conclusion

In summary, we prepared a facile, cost‐efficient, and environmentally friendly method to obtain high‐performance embedded FTEs with LS‐EFD microscale 3D printing. The final FTEs manufactured by this technique has excellent photoelectric performance with an *R*_s_ of 6 Ω sq^−1^ and *T* of 92.85% at a wavelength of 550 nm. The embedded metal structure gives our FTEs excellent mechanical, chemical, and environmental adaptability. The change rate of *R*_s_ is lower than 16%, 13.8%, and 3.85%, respectively, after 1000 bending cycles, 72 h of chemical corrosion, and 72 h of damp heat. These excellent metrics proved that the embedded metal mesh has good stability in many practical applications including flexible electronics, transparent electric heating, and sensors. We used the material for thermally driven 4D printing structure and resistive strain sensor were prepared showing good practical application effects. Hence, our FTEs can be used for facile, environmentally friendly, and high‐throughput manufacturing. This can lead to a strategy for low‐cost and green manufacturing of flexible optoelectronic devices.

## 4 Experimental Section

### Materials

All the solvents were purchased from Sinopharm Co., Ltd. (China). The PDMS (Dow Corning SYLGARD 184) and curing agent were purchased from Dow Corning (USA). Nanoconductive silver paste (NT‐TL20E) and stretchable silver paste (ST201S1G01A) were purchased from the Beijing NanoTop Electronic Technology Co., Ltd. (China). The PET was purchased from Dongguan Dejin Plastic Insulation Material Co., Ltd. (China). Ecoflex 00‐30 was purchased from Smooth‐On Store (USA).

### Fabrication of the FTE with Embedded Metal Mesh

A layer of PDMS (ratio of the PDMS elastomer and curing agent: 10:1) was first spin‐coated on the PET substrate. The metal mesh was next printed using an LS‐EFD microscale 3D printing technology, and the line width and pitch of the metal mesh was controlled by adjusting the LS‐EFD process parameters. The embedded metal mesh was then conductively treated at 130 °C for 60 min. Finally, FTEs was prepared with an embedded metal mesh.

### Characterization

Optical images of the metal mesh were captured through an optical microscope (DSX510, OLYMPUS, Japan; Phenix MC‐D500U(C), China). The microstructure and cross‐section of the metal mesh was characterized via field‐emission SEM (MERLIN Compact, Zeiss, Germany). The printing process of the embedded metal mesh was captured by a high‐speed camera (i‐SPEED221, IX Cameras, UK). The embedded metal mesh was conductivity‐treated in a vacuum drying oven (DHG‐903385‐III, Shanghai Shengke Instrument Equipment Co., Ltd., China). The optical transmittance of the metal mesh was measured with a UV–vis spectrophotometer (UV‐6100, Metash, China). The heating performance of FTE was captured by infrared thermal imaging (TG165, FLIR Systems. USA). The *R*_s_ of the FTE is measured by a milliohmmeter (AT516, Applent Instruments Co., Ltd., China). The damp heat test of embedded metal mesh was measured by constant temperature and humidity test chamber (Dongguan Seth Testing Equipment Co., Ltd., China). The bending fatigue test was completed via a self‐developed test system.

## Conflict of Interest

The authors declare no conflict of interest.

## Supporting information

Supporting Information

Supplemental Movie 1

Supplemental Movie 2

Supplemental Movie 3

Supplemental Movie 4

Supplemental Movie 5

## Data Availability

Research data are not shared.
