# Supplementary material for: Directly Printed Embedded Metal Mesh for Flexible Transparent Electrode via Liquid Substrate Electric‐Field‐Driven Jet
Source: Adv Sci (Weinh). 2022 Mar 1;9(14):2105331. doi: 10.1002/advs.202105331 (PMC9108624; doi:10.1002/advs.202105331)
Supplement: Supplementary file 1 — Supporting Information [file ADVS-9-2105331-s004.pdf]

ADVANCED SCIENCE Open Access

Supporting Information

for *Adv. Sci.*, DOI 10.1002/advs.202105331

Directly Printed Embedded Metal Mesh for Flexible Transparent Electrode via Liquid Substrate Electric-Field-Driven Jet

Zhenghao Li, Hongke Li, Xiaoyang Zhu, Zilong Peng, Guangming Zhang, Jianjun Yang, Fei Wang, Yuan-Fang Zhang, Luanfa Sun, Rui Wang, Jinbao Zhang, Zhongming Yang, Hao Yi and Hongbo Lan**

Supporting information

Directly Printed Embedded Metal Mesh for Flexible Transparent Electrode via Liquid Substrate Electric-Field-Driven Jet

Zhenghao Li#, Hongke Li#, Xiaoyang Zhu#, Zilong Peng, Guangming Zhang, Jianjun Yang, Fei Wang, Yuan-Fang Zhang, Luanfa Sun, Rui Wang, Jinbao Zhang, Zhongming Yang, Hao Yi, and Hongbo Lan**

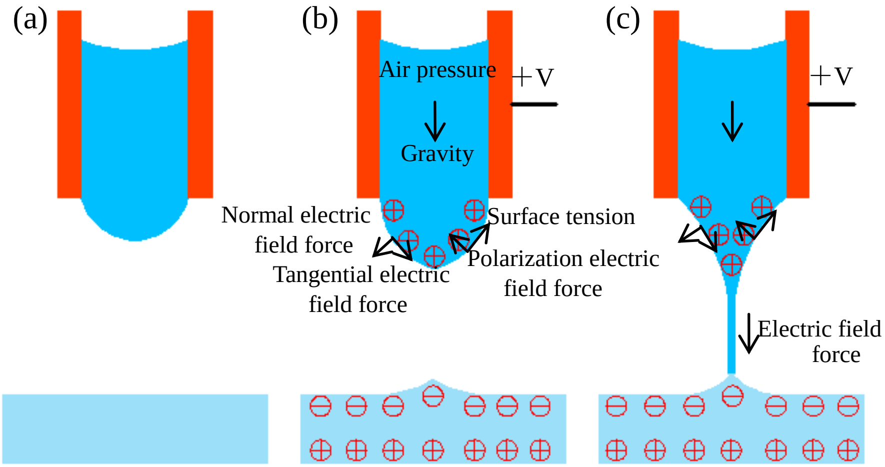

Figure S1. Schematic diagram of the principle of LS-EFD microscale 3D printing

A numerical simulation model was created in a 2D space using COMSOL multiphysics simulation software, as shown in Figure S2. The red area represents the printing nozzle, a positive voltage was applied to the print nozzle, i. e., the boundary is the potential boundary condition; The blue area represents the PDMS substrate with a thickness of 40 mm, the printing substrate boundary (e, f, g, h) is the suspension potential boundary condition; The gray area represents the printing material (conductive silver paste); The white areas represent the surrounding air. Boundary a-d is the grounding boundary condition, i. e., potential V=0; The initial value of the potential in all solution domains is 0, i.e. V = 0. The 2D simulation model is meshed with the maximum cell size set to 0.05mm and the minimum cell size set to 0.001mm, resulting in a complete mesh of 30548 domain cells and 1416 boundary cells.

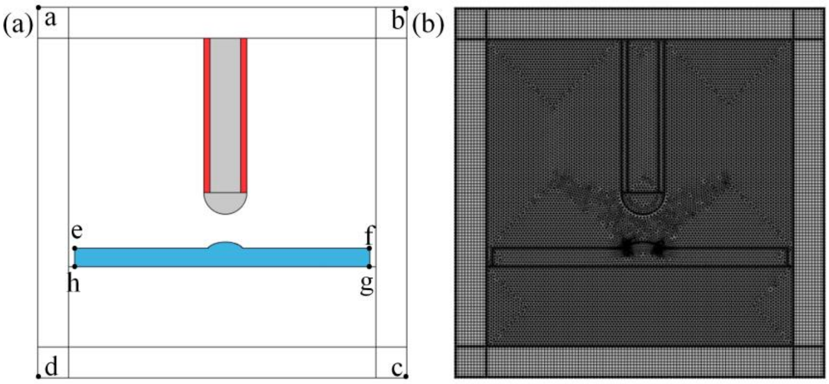

Figure S2: (a) the geometric model, (b) the mapped meshing

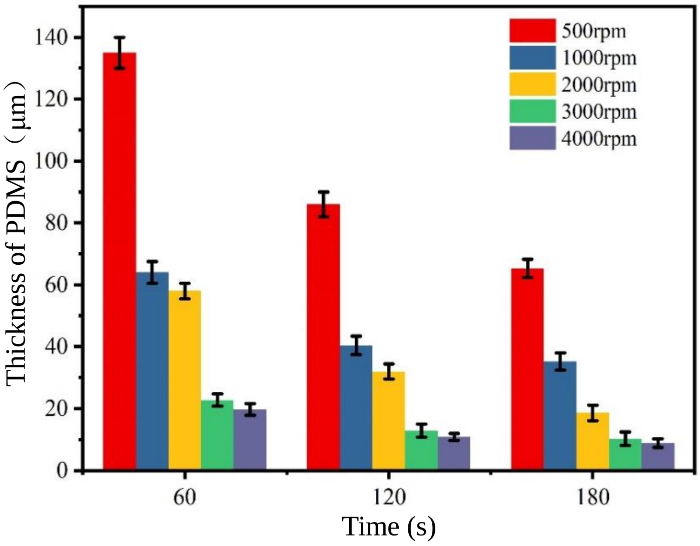

Figure S3. Thickness of PDMS under different spin coating parameters.

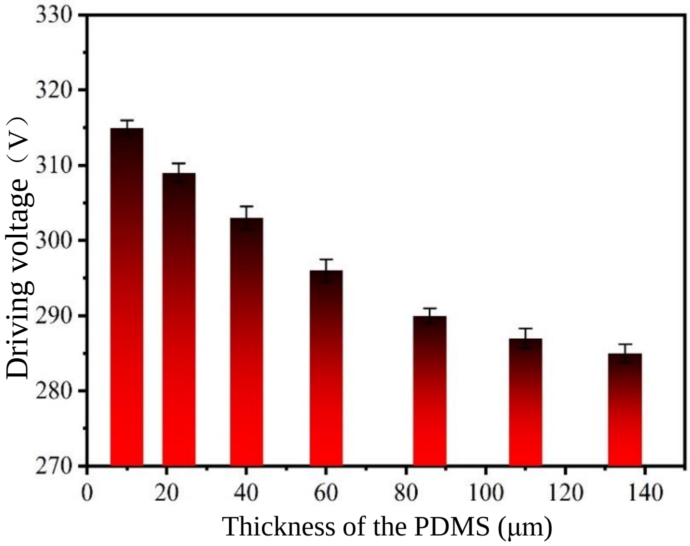

Figure S4. Driving voltage under different PDMS liquid film thickness

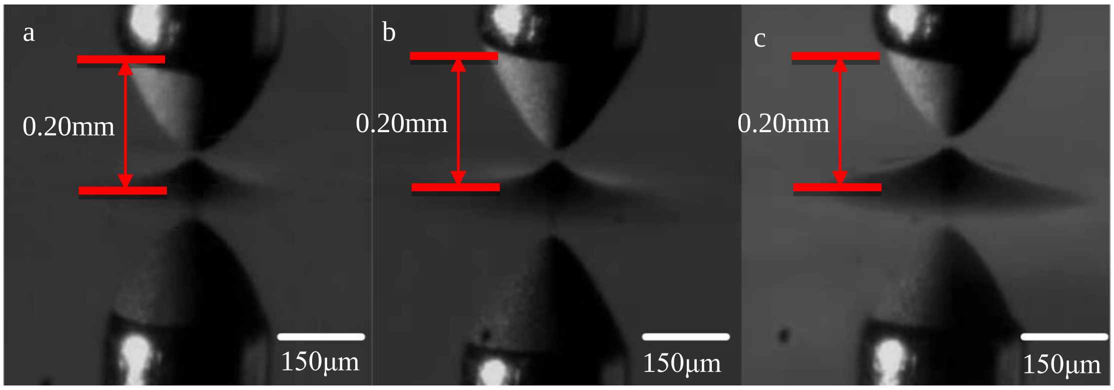

Figure S5. Corresponding structure of liquid needlepoint under different PDMS thickness: a) 8.5 μm; b) 40 μm; c) 60 μm.

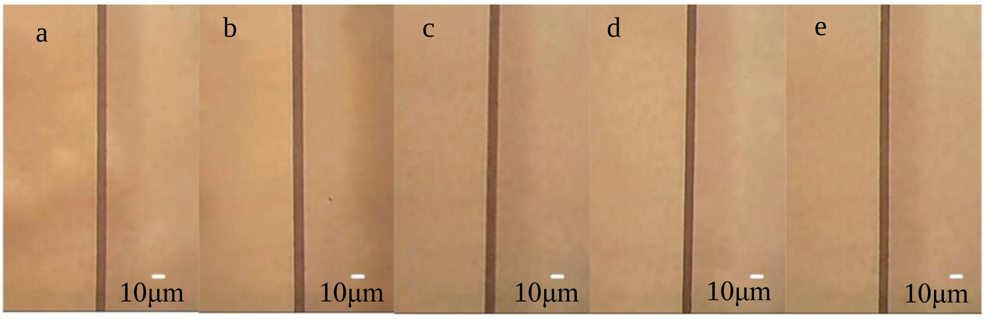

Figure S6. Microstructure of silver wires under different PDMS liquid film thickness: the corresponding PDMS thickness of a-e is 8.5 μm, 40 μm, 60 μm, 86 μm, 110 μm, respectively.

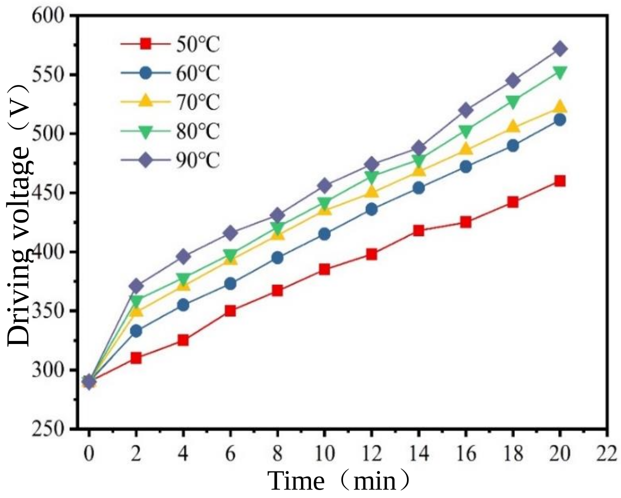

Figure S7. The minimum stable driving voltage corresponding to different curing degrees of PDMS.

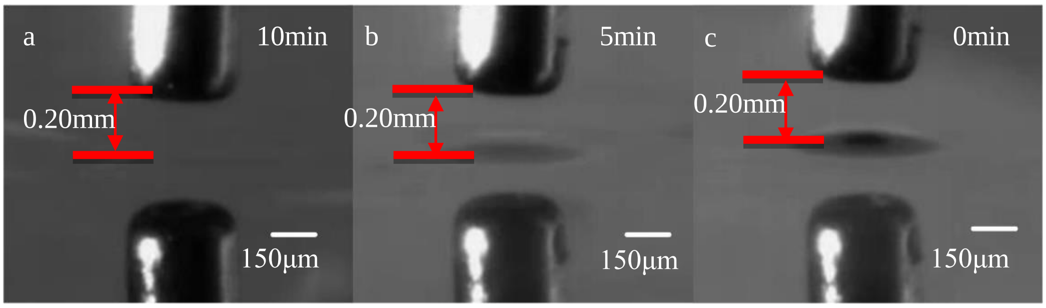

Figure S8. The size of liquid needlepoint structure formed by PDMS under different curing degrees: a) curing at 80℃ for 10 min; b) curing at 80℃ for 5 min; c) curing at 80℃ for 0 min.

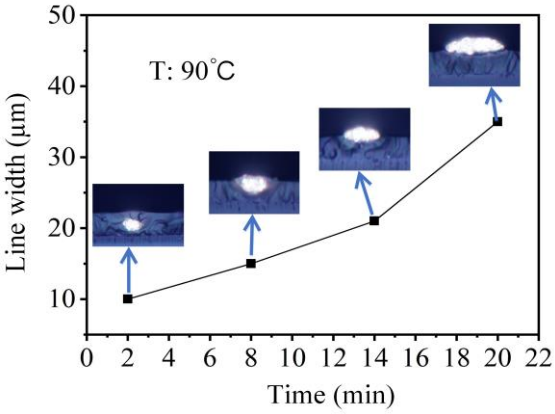

Figure S9. The embedment state of silver wire in PDMS under different curing degrees

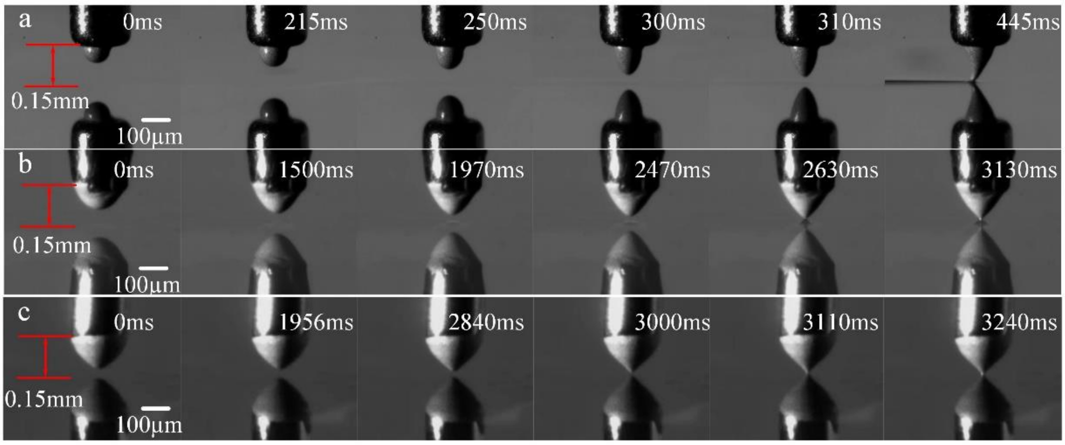

Figure S10. The jet printing process of PDMS in different states: a) Fully cured PDMS substrate; b) Semi-cured PDMS substrate; c) Uncured PDMS substrate.

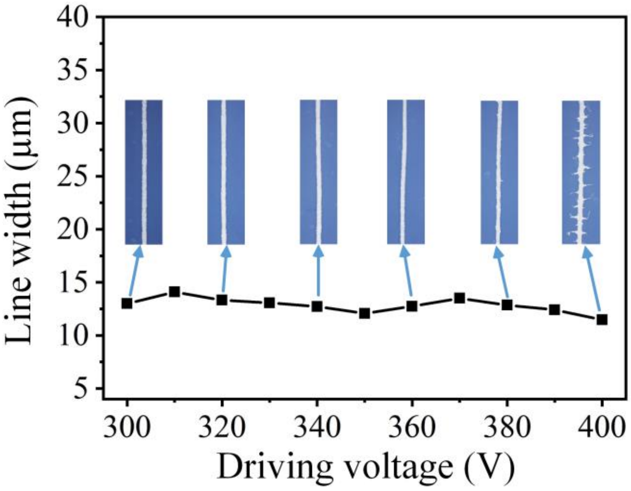

Figure S11. Microstructure of silver wires under different driving voltages.

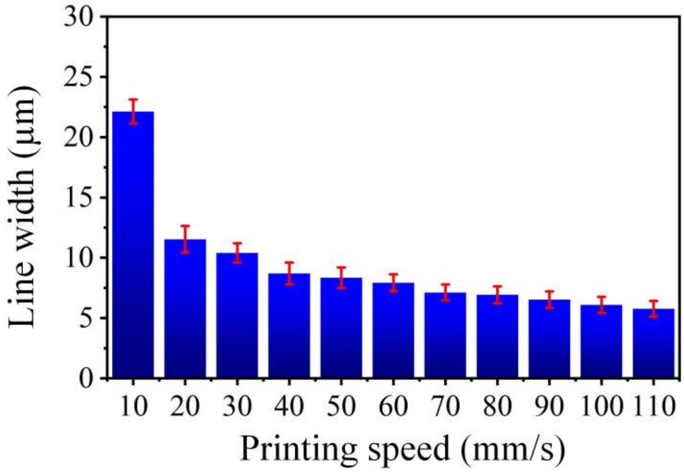

Figure S12. Silver line width at different printing speeds

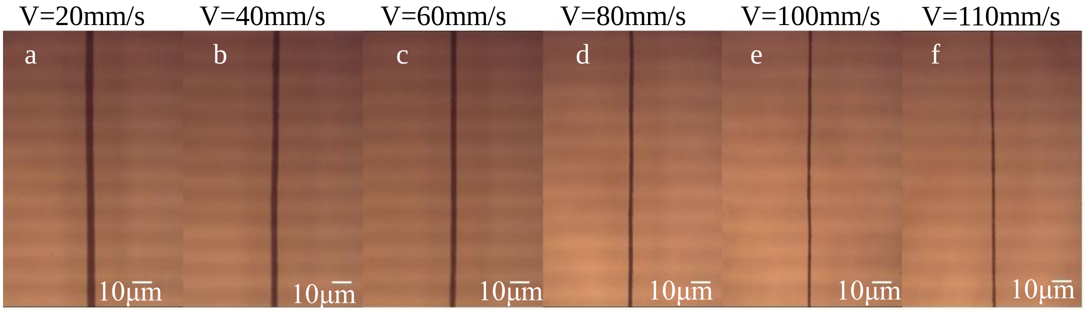

Figure S13. Micromorphology of silver wires at different printing speeds

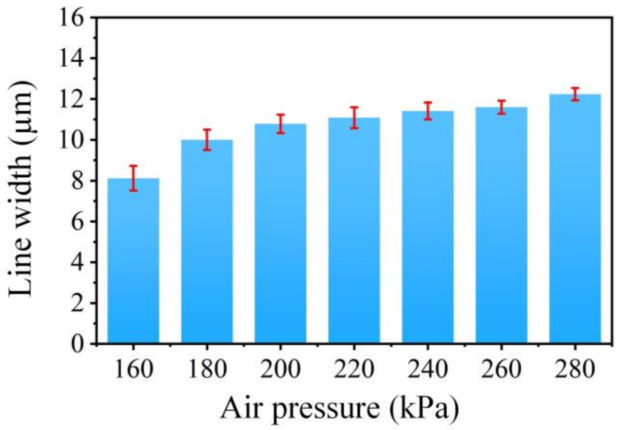

Figure S14. Silver line width at different air pressure

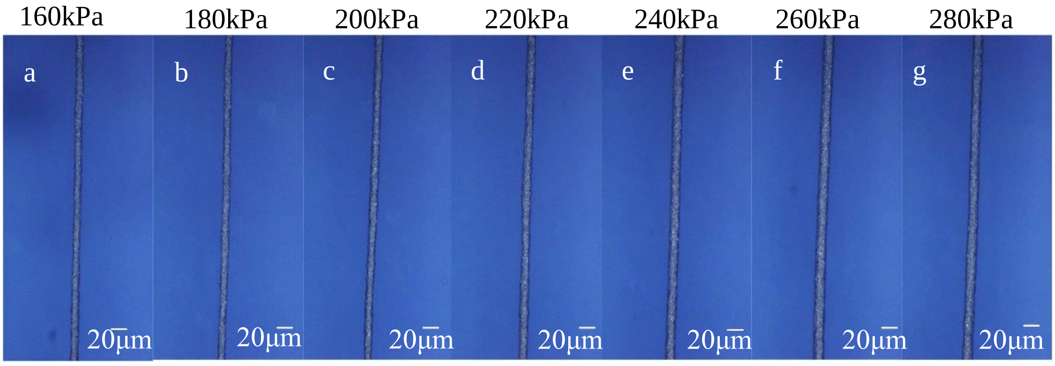

Figure S15. Micromorphology of silver wires at different air pressure

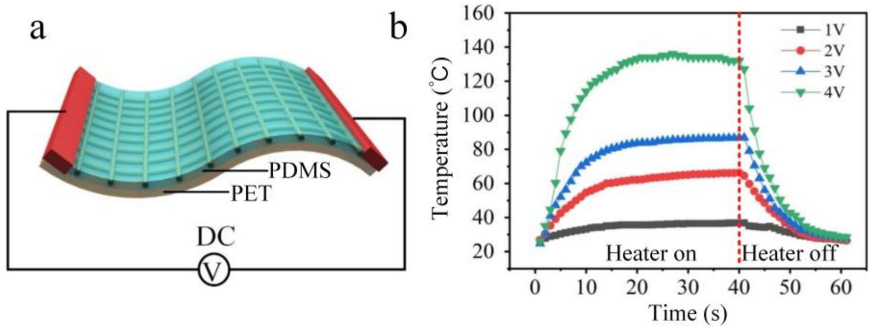

Figure S16. Heating characterization of the FTE: a) The heating schematic diagram of the FTE; b) The thermal response diagram of FTE

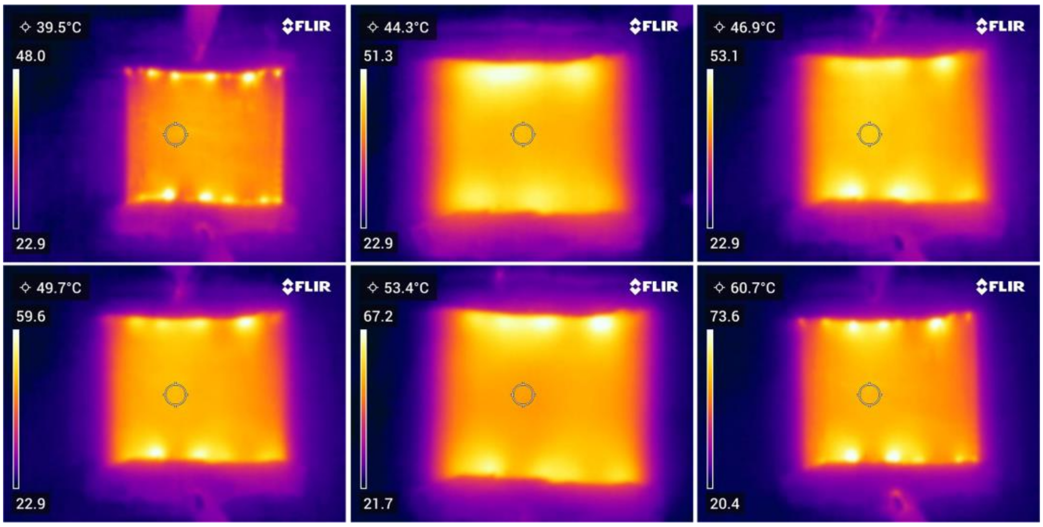

Figure S17. A temperature distribution image of the FTE at 2V DC voltage

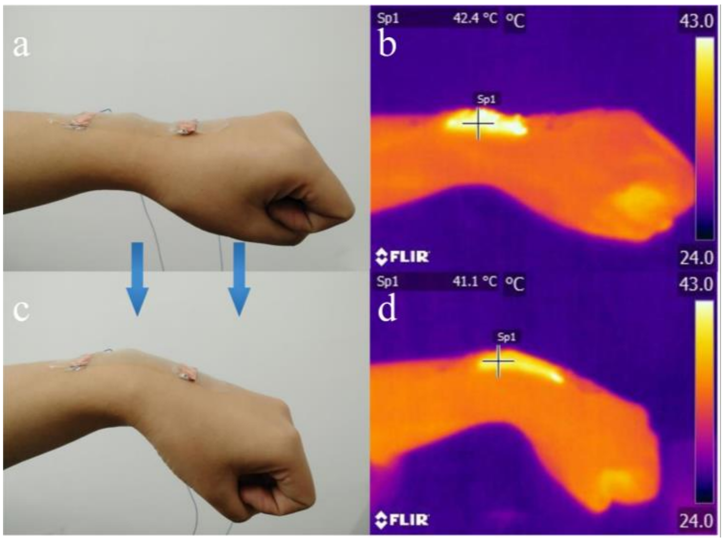

Figure S18. Application of hyperthermia in human wrist
